# Effect of Addition of Pectins from Jujubes (*Ziziphus jujuba* Mill.) on Vitamin C Production during Heterolactic Fermentation

**DOI:** 10.3390/molecules25112706

**Published:** 2020-06-11

**Authors:** Alessia Fazio, Chiara La Torre, Maria Cristina Caroleo, Paolino Caputo, Roberto Cannataro, Pierluigi Plastina, Erika Cione

**Affiliations:** 1 Department of Pharmacy, Health and Nutritional Sciences, Department of Excellence 2018–2022, University of Calabria, Edificio Polifunzionale, 87036 Rende (CS), Italy; latorre.chiara@libero.it (C.L.T.); mariacristinacaroleo@virgilio.it (M.C.C.); r.cannataro@gmail.com (R.C.); pierluigi.plastina@unical.it (P.P.); erika.cione@unical.it (E.C.); 2 Department of Chemistry and Chemical Technologies, University of Calabria, 87036 Rende (CS), Italy; paolino.caputo@unical.it

**Keywords:** jujube, harvesting stage, pectin, methoxylation degree, kefir, vitamin C, heterolactic fermentation

## Abstract

Soluble fibers, including pectins from apple and lemon, are commonly used as prebiotic and to prepare functional foods. The present study aimed to investigate the physicochemical and functional properties of pectins extracted from jujubes (*Ziziphus jujuba* Mill.). Pectins were extracted from jujubes at three stages of harvesting and characterized by FTIR and SEM analyses. Whole milk inoculated with kefir grains was supplemented by 0.25 mg·mL^−1^ of pectins. The pH value and vitamin C content were evaluated after 24 and 48 h of fermentation. Pectins from jujubes at the first harvesting stage (PJ1K) showed the lowest methoxylation degree. The addition of pectins enhanced the production of vitamin C during heterolactic process. This result was found to depend on jujube harvesting stage as PJ1K stimulated the growth of yeasts in kefir grains yielding to the highest amount of vitamin C (0.83 ± 0.01 µg·mL^−1^) compared to other samples (0.53–0.60 µg·mL^−1^) at 24 h. Lactic acid bacteria diminish pH rapidly with respect to control (4.13 ± 0.05), according to the stage of maturation, reducing its initial value by 38.3% in PJ1K. Besides being an excellent prebiotic, pectins from jujubes could be used to enrich kefir with vitamin C.

## 1. Introduction

Jujube is the fruit of the Ziziphus plant that is widespread in China and other regions of Asia and Australia as well as in the Mediterranean basin. In China, its cultivation area has reached the remarkable amount of more than 1.5 million ha, with an annual fruit production of 400,000 tons and an export of US $5 million [1]. Jujube fruits have been reported to have multiple biological functions due to the presence of bioactive compounds including phenolics, triterpenoids, saponins, and non-starch polysaccharides. These bioactive compounds have been used in the prevention and treatment of human diseases, such as inflammatory diseases, cancers, liver damage, and wound healing [2,3,4,5]. In particular, its content in dietary fiber contributes to consider it a healthy choice of food [6]. Pectins are polysaccharide derivatives representing the major component of the primary cell walls and middle lamella of plant tissues, where they function as hydrating agents and cementing material for the cellulosic network [7]. In the pharmaceutical field, they find a variety of applications as a tool to reduce blood cholesterol and to prevent various types of cancers [7], while they are used in the food industry as gelling agent and stabilizer [8]. Pectins are food components that have beneficial effects on human health as they possess prebiotic nature and nutraceutical properties and act as a delivery vehicle for probiotics, and for these reasons they find positive utilization in promoting health by modulation of intestinal microbiota [9]. It has been reported that the addition of prebiotics, such as inulin [10] or fructooligosaccharides (FOS) [11], to fermented milks influences physicochemical and sensory properties, as well as microbiological growth, vitamins, and organic acid formation. In this context, kefir is a fermented dairy beverage with an alcoholic flavor, easy to digest, and rich in probiotic microbiota [12].

Kefir comes from the Caucasus Mountains, and is produced by incubating milk with kefir grains [13] or freeze-dried concentrated cultures that are even considered to lead to a more uniform and stable product, thus guaranteeing its commercial value [14,15]. The most common microorganisms present in kefir are lactic acid bacteria (*Lactobacillus*, *Leuconostoc*, *Streptococcu,* and *Lactococcus* ssp), fermenting yeasts (*Kluyveromyces*, *Saccharomyces*), and acetic bacteria [15,16]. Lactic acid bacteria (LAB) can be responsible for both homofermentation that produces lactic acid and heterofermentation leading to acetic acid, ethanol, carbon dioxide, formic acid, and lactic acid [17]. This beverage is a recognized probiotic dairy product [18]: Its beneficial properties are well known and currently there is a positive trend for its consumption [19]. Kefir is an example of probiotic mixture of bacteria and yeasts [20], which take part to complex interactions contributing to the symbiosis equilibrium and can influence product characteristics and quality [21]. In this regard, it is useful to highlight that kefir also lends itself to “natural” vitamin fortification [12]. Interestingly, Di Matteo et al. [22] have reported that the ascorbic acid content is associated with the expression of genes involved in pectin degradation. Although synthetic vitamin C can be readily added to food, there is an increasing awareness and resistance among the consumers to the use of synthetic additives in food materials and a tightening of the legislation on food and drink artificial fortification [23]. These results prompted us to explore the possible influence of pectins on the synthesis of vitamin C by the kefir microorganisms. Although the effect of the addition of apple- and lemon-soluble fibers to kefir on rheological, microbiological, and sensorial properties has been reported [24,25], to the best of our knowledge, there are no data available in the literature about the effect of pectin addition on chemical composition of milk kefir. In order to obtain functional and biofortified foods, the objective of this study was to evaluate the effect of the addition of pectins extracted from Chinese dates (jujubes) (*Ziziphus jujuba* Mill.) on vitamin C production during kefir heterolactic fermentation in whole milk.

## 2. Results and Discussion

### 2.1. Extraction of Pectins

In order to valorize jujube fruits, chemical and morphological characterization of jujube pectins was assessed and, as a result, the effects of three different stages of jujube harvesting on methoxylation degree of extracted pectins were assessed. The diverse properties of pectins depend on the food matrix from which they are extracted and on the isolation method, all factors that influence the methoxylation degree.

Extraction of pectins from jujube fruits was performed by using citric acid under reflux. The choice of this acid instead of diluted mineral acids, such as sulfuric, hydrochloric, and nitric acids, is due to the advantages it offers as regards the environmental impact and the economic aspect [26]. Pectin recoveries from jujubes of the first, second, and third harvesting were 48 ± 4 mg·g^−1^ (4.8% yield), 37 ± 3 mg·g^−1^ (3.7% yield), and 21.9 ± 0.6 mg·g^−1^ (2.2% yield), respectively. These results showed that the pectin content changed markedly as jujube fruit ripened: Extraction yield was halved at the third ripening stage (2.2%) compared to that of the first harvest (4.8%).

In line with our results, it has been reported that pectin content from jujubes of different cultivars, extracted by dissimilar methods [27] ranged from 3 to 7.5%. This highlights that the jujube fruit could represent a promising alternative source of pectins, in particular at the first stage of maturation.

### 2.2. FTIR Spectroscopic Analysis

**Figure 1:**
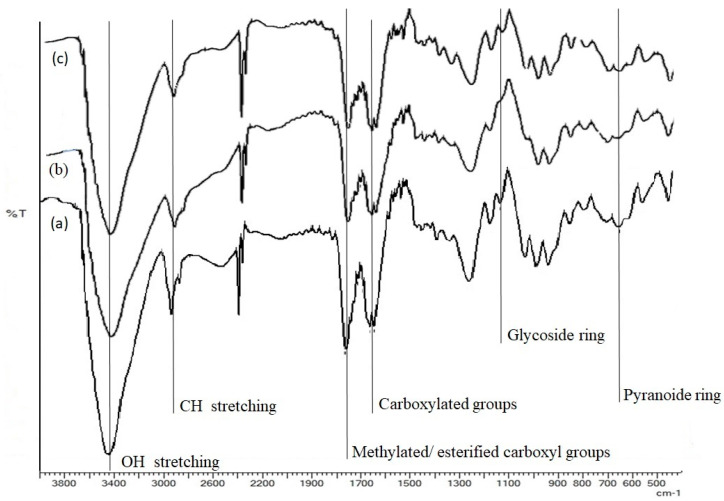
FTIR spectra of pectins in the 400 to 4000 cm^−1^ region. Spectra of pectins from jujubes (PJ) of first harvesting, PJ1 (a), second harvesting, PJ2 (b) and third harvesting, PJ3 (c).

The FTIR spectra of pectins from jujubes (PJ) of first, second and third harvesting (PJ1, PJ2, PJ3, respectively) are reported in Figure 1. Typical bands appeared in the spectra, as it is expected for pectin molecules, which prove that the extracted substances were pectins; the bands characterizing the structure did not show significant differences, except for their relative absorption intensities, at the three stages of harvesting. The characteristic bands, around 3640−3680 cm^−^^1^, are generated by OH stretching, due to inter- and intramolecular hydrogen bonding of the galacturonic acid backbone. The band at around 2924−2928 cm^−^^1^ is due to CH stretching and bending vibrations of galacturonic ring. Another important region is the fingerprint region 1750−1350 cm^−^^1^, where the bands allowed the detection of esterified (methylated) vs. unesterified functional groups. The extracted pectins from jujubes of all harvesting periods revealed in this spectral region two bands at 1748 cm^−^^1^ and 1647 cm^−^^1^. The first one is due to the C=O, ester carbonyl groups’ stretching, and the second one is generated by COO^-^ carboxylate ion stretching [28]. In the so-called “fingerprint region” (1300 to 900 cm^−^^1^) [29], the band at 1425 cm^−1^ is originated from the symmetric and asymmetric stretching vibration of the methyl group in ester, while bending vibration of OH group in pyranose ring generated the band at 1235–1230 cm^−1^. The bands occurring at 900–910, 1008–1004, and 1040 cm^−1^ were generated from C−C stretching, C−O−H deformation, and asymmetric C−O−C stretching vibration. In addition, the weak band at 840 cm^−1^ indicated the ring vibration of α-glycosidic linkage. These bands were characteristic for pectins [30].

### 2.3. Scanning Electron Microscopy

**Figure 2:**
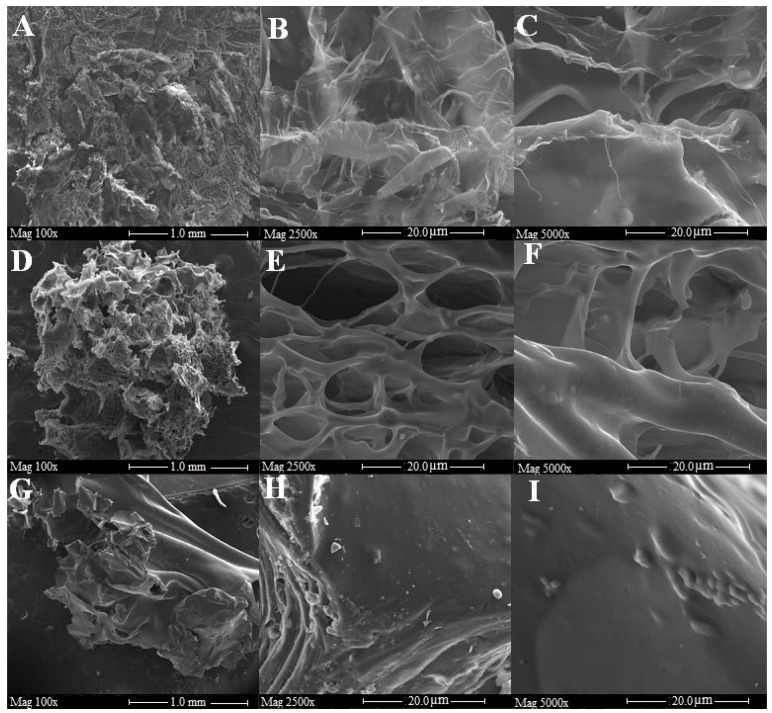
Scanning electron images of PJ1 (**A**–**C**), PJ2 (**D**–**F**), and PJ3 (**G**–**I**). The magnification of (**A**,**D**,**G**) is equal to 100× with a scale of 1 mm; magnification of (**B**,**E**,**H**) is equal to 5000× with a scale of 20 μm; magnification of (**C**,**F**,**I**) is equal to 2500× with a scale of 20 μm.

The structural characterization of pectin samples was performed by scanning electron microscopy (SEM) equipped with wavelength dispersive spectroscopy (WDS). SEM images (Figure 2A–I) with different magnifications, providing visual surface morphology features of dried pectin particles, showed that different degrees of fruit ripeness could affect the microstructures of pectins. It can be seen that the surface of PJ1 was compact (Panel A) with the presence of micro-cracks and elongated holes of the order of hundreds of microns. In the panels B and C with greater magnification (2500× and 5000×, respectively) irregular lamellar structure with different thicknesses, which was closely packed, is visible. Panel D shows that pectins from J2 have rough texture and plenty of surface irregularities. At higher magnifications (panels E–F), it is possible to see a very porous structure with intercommunicating cavities of dimensions up to the order of tens of microns. Panel G shows a dense, amorphous structure which was soft and could curl easily, devoid of porosity and cavity. At high magnifications, it can be seen that the surface of PJ3 was dense and stratified (panel H) with shallow pitting (panel I). Then, it is possible to state that the three samples have a different degree of porosity and compactness, ranging from the extremely porous (panels D, E, F) to the more compact and non-porous (panels G, H, I), passing through an intermediate situation (panels A, B, C).

The Wavelength Dispersive X-ray Spectroscopy (WDS) analysis was performed in order to determine the variation of the chemical composition in the pectin matrix during the maturation period under study.

Table 1 presents the results of the WDS measurements in the pectin samples. The data were an average of three measurements in various particles of each sample. WDS analysis results revealed that the main element in all samples is the carbon followed by the oxygen, while nitrogen is a negligible percentage, whose content ranged from 1.24% in PJ1 to 1.92% in PJ3. The carbon percentage increased from 46.17 (PJ1) to 69.87 (PJ3), while the oxygen percentage increased at the second stage of harvesting (12.93%) and remained constant at the third stage. The findings demonstrated that the ripening process had effect on the morphological characteristics of jujube pectins [31]. Probably, during the harvesting period under study, as the maturation proceeded, the color of the jujubes underwent the increase in redness and the loss of greenness, as shown in Figure 1, without affecting the texture of fruit, which retained its hardness. These aspects confirmed that softening process, caused by the degradation and solubilization of cell wall polysaccharides, had not yet begun at the third harvesting period; the depolymerization of the homogalacturonan backbone by higher polygalacturonase [32] was not yet started, as confirmed by the increase of the degree of polymerization in PJ3, while the chain length, the branch amounts, and aggregate sizes increased, as evidenced by the rinsed percentage of carbon in PJ3.

### 2.4. Determination of Esterification Degree

**Figure 3:**
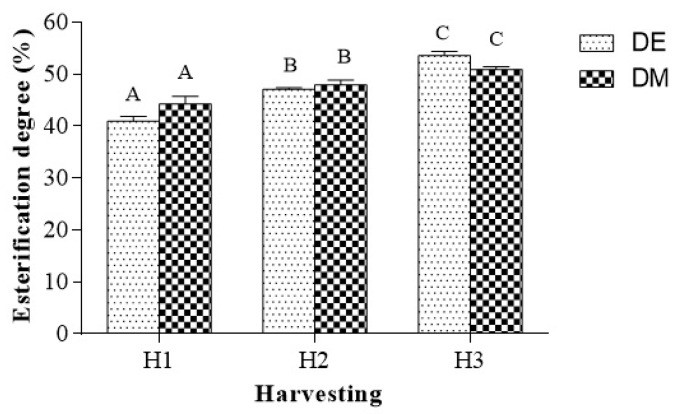
Effects of harvesting (H1, H2, and H3) on esterification degree determined by titrimetric (esterification degree determined by titration method, DE) and instrumental methods (esterification degree determined by instrumental FTIR method, DM). Error bars represent standard deviation (n = 3). Values with the same letter are not significantly different, while values with different letters are significantly different (DE: H1 vs. H2, ** *p* < 0.01, H1 vs. H3, **** *p* < 0.0001, H2 vs. H3, ** *p* < 0.01; DM: H1 vs. H2, ** *p* < 0.01, H1 vs. H3, ** *p* < 0.01, H2 vs. H3, not significant, ns).

The degree of methoxylation of pectins is an important property in food processing systems due to its effect on functional properties such as gelling and solubility [33]. Moreover, it allows classifying pectins into two types: High-methoxyl (HM) form, characterized by a percentage of esterified groups greater than 50%, and low-methoxyl (LM) form, in which the percentage of esterified carboxyl groups was less than 50%. The degree of esterification of pectins from jujubes at three stages of harvesting (H1, H2, and H3) was determined by both classical titrimetric method (esterification degree determined by titration method, DE) and instrumental FTIR method (esterification degree determined by instrumental FTIR method, DM) (Figure 3). The degree of esterification, regardless of the method used, exhibited a significant increase as maturation proceeds. The titrimetric values for DE of pectins increased from 40.8% in H1 to 53.6% in H3. The instrumental technique, based on FTIR spectroscopy, provided slightly higher values for H1 (44.3%) and slightly lower values for H3 (50.9%), compared to the corresponding results of the titrimetric analysis. In both cases, statistical analysis showed that pectin degree of methoxylation from H1 jujubes is significantly lower than others. Each sample was analyzed in triplicate by both titrimetric and instrumental methods and the results are given as means ± standard deviation.

### 2.5. Evolution of pH and Kefir Weight during Heterolactic Fermentation

**Figure 4:**
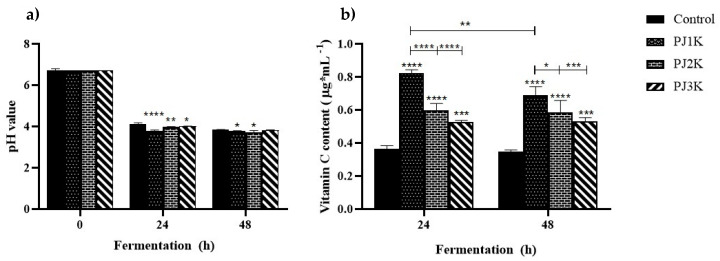
Evolution of pH (**a**) and vitamin C content (**b**) during fermentation, in control and pectin-supplemented kefirs. PJ1K: Kefir added with PJ1. PJ2K: Kefir added with PJ2. PJ3K: Kefir added with PJ3. Values represent the mean of three independent experiments (each done in triplicate) ± standard deviation. Asterisks on the bars indicate that mean values were statistically different from the control, while asterisks on the dashes indicate differences among different treatments or the same treatment at different time points (* *p* < 0.05, ** *p* < 0.01, *** *p* < 0.001, and **** *p* < 0.0001).

At time 0 h, before inoculation with kefir grains, the average whole milk pH was 6.7. As the fermentation progressed, the pH of kefir-added milk (control) decreased as a result of the growth of lactic acid bacteria and lactic acid production [34,35]: The initial value of pH was reduced by 38.3% up to the average value of 4.13 ± 0.05 after 24 h and by 42.7% to 3.84 ± 0.05 after 48 h (Figure 4). In pectin-supplemented kefirs (PJ1K, PJ2K, PJ3K), the pH value was 3.73 ± 0.02 for PJ1K, 3.97 ± 0.02 for PJ2K, and 4.01 ± 0.02 for PJ3K, after 24 h of fermentation (day 0). Significant higher acidity (**** *p* < 0.0001) resulted in PJ1K, in PJ2K (** *p* < 0.01), and in PJ3K (* *p* < 0.05). After 48 h of fermentation, pH was still low in PJ1K (* *p* < 0.05) and PJ2K (* *p* < 0.05). Lastly, the results showed that the addition of the pectins from jujubes at three different harvesting periods influenced the pH of supplemented kefirs with respect to the control, obtaining statistically significant decline of pH in kefir-supplemented pectins especially in PJ1K after 24 h of fermentation (*p* < 0.0001).

Kefir grains are a cluster of microorganisms that make a complex microbiota which coexists in an exopolysaccharide matrix called kefiran, produced by *Lactobacillus kefiranofaciens*. As the cell growth and kefiran production rate is increased when *L*. *kefiranofaciens* is grown under the conditions created by yeasts [36], the increase in weight of the kefir grains was taken into account to confirm the amplified activity of the bacteria, although it is not correlated with the production of vitamin C. The data obtained (Table 2) showed that the greatest weight gain was recorded for the PJ1K, in which the kefir grains are about 1.5 times heavier than the starting grains, witnessing the greatest activity of *L*. *kefiranofaciens* stimulated by the yeast in kefir sample added with pectins from green jujubes.

### 2.6. Vitamin C Content during Heterolactic Fermentation

Recently, functional and bio-fortified foods are receiving great interest around the world as demonstrated by their huge market size. Functional foods are a fashion trend for the industrialized countries to keep health status and to prevent chronic disease, while in low-income countries, bio-fortified foods are a necessity to reach the health status and to prevent malnutrition and infectious diseases [37]. Among functional foods, fermented ones had longer storage life and improved nutritional values compared to their unfermented equivalents, making this form of food processing a popular technique. Kefir has a low production cost, can be produced at home, and can be easily incorporated into the diet. During the kefir fermentation process, approximately 30% of the lactose from milk is hydrolyzed by the β-galactosidase enzymes, turning lactose into glucose and galactose. Furthermore, bacteria present in kefir convert glucose into lactic acid, which causes pH reduction and increases in consistency. In this context, kefir is a good option for lactose-intolerant individuals [38]. In addition, it is known that the fermentation induced by kefir has significant influences on vitamin content [12]. We found that whole milk does not contain vitamin C and, therefore, it has been used as a raw material in which to carry out all the experiments in order to better verify any positive influence of pectins on the production of vitamin C.

Our study showed that the addition of pectins enhanced the production of vitamin C; the content of vitamin C was quantified in the control and pectin-supplemented kefirs during fermentation (24 h and 48 h) by HPLC (Figure 4). In the control, this vitamin reached a level of 0.36 ± 0.01 µg·mL^−1^ at 24 h of fermentation, while in all pectin-supplemented kefirs a higher content of vitamin C was detected: Ascorbic acid content was 0.83 ± 0.01 µg·mL^−1^ in PJ1K, 0.60 ± 0.02 µg·mL^−1^ in PJ2K, and 0.53 ± 0.01 µg·mL^−1^ in PJ3K at 24 h of fermentation. All the results obtained were significantly different compared to the control (**** *p* < 0.0001). In particular, the addition of pectins from H1 jujubes (PJ1K) resulted in a quantitatively higher vitamin C level compared to all the other treatments (PJ2K and PJ3K), after 24 h fermentation (**** *p* < 0.0001). The results related to the production of vitamin C after 24 h of fermentation were in agreement with the pH values reached in PJ1K, after the same time: PJ1 kefir with the highest vitamin level was also the one with the most acidic pH value (3.73 ± 0.02). After 48 h fermentation, the content of vitamin C in PJ1K decreased compared to that obtained after 24 h fermentation, reaching a final value of 0.69 ± 0.04 µg·mL^−1^ at the end of the period studied (** *p* < 0.01), but, in any case, it was significantly higher than the control (**** *p* < 0.0001), as well as than PJ2K (* *p* < 0.05) and PJ3K (*** *p* < 0.001).

In PJ2K and PJ3K, vitamin C content remained constant compared to that obtained after 24 h fermentation. Also, kefir samples were added with higher amount of pectins, but after fermentation the content of vitamin C was not increased (data not shown), if compared to the content found in kefir sample supplemented by 0.25 mg·mL^−1^ of pectins, which represents the maximum amount used in all experiments. The results showed that the vitamin C content, which was higher in kefir supplemented with pectins from green jujube, was correlated to the pectin methoxylation degree: The production of vitamin C was greater if the methoxylation degree was lower. This correlation would seem to support the hypothesis [39] according to which the yeasts present in kefir grains can assimilate galactose and may produce vitamin C (0.36 ± 0.01 µg·mL^−1^ in the control). In fact, during fermentation an interaction between yeasts and LAB occurs, suggesting that the yeasts could provide vitamins, amino acids, and growth factors for bacteria, while the bacterial end products could be used by the yeasts as an energy source. Yeasts stimulated the LAB through production of carbon dioxide, pyruvate, propionate, and succinate, and some LAB release galactose into the medium as a by-product of lactose metabolism, which may be assimilated by yeast. In pectin-supplemented kefir, galactose is not only that produced by LAB, but also that deriving from pectins, which, especially when characterized by a lower degree of methoxylation, are more easily hydrolyzable by enzymes and provide the individual units of galactopyranosyluronic acid as a substrate for yeasts. In fact, the hydrolysis of the glycosidic bonds in the main pectin backbone occurs more easily if the monosaccharide units are demethylated, as evidenced by the hydrolytic activity of the polygalacturonases. These actions would explain why the addition of pectins caused an increase in the production of vitamin C (0.83 ± 0.01 µg·mL^−1^ in PJK1) during heterolactic fermentation; this production decreases proportionally as the degree of methoxylation increases (0.53 ± 0.01 µg·mL^−1^ in PJK3). Also, it is possible to establish a correlation between increased production of vitamin C and decrease in supplemented kefir pH during fermentation, compared to the control. Vitamin C production by yeasts would seem to enhance the growth of lactic acid bacteria, which also produce lactate. Then, lactic acid bacteria diminish pH rapidly (3.73 ± 0.02 for PJ1K) with respect to control (4.13 ± 0.05), while lactate accumulation until this production is inhibited and components that are responsible for the flavor, such as acetaldehyde, are produced along with aromas of fermented milk.

**Figure 5:**
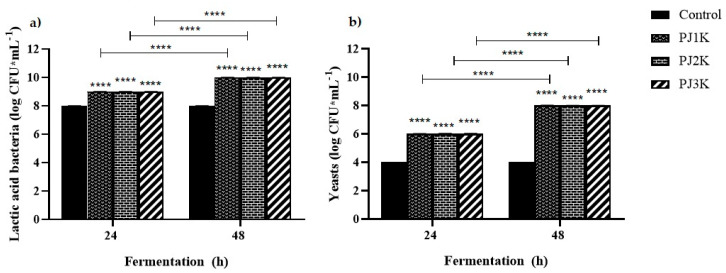
Cell counts of lactic acid bacteria (**a**) and yeasts (**b**) populations in kefir samples supplemented with (or without, used as control) 0.25 mg·mL^−1^ of PJ1 (PJ1K), PJ2 (PJ2K), and PJ3 (PJ3K) during fermentation. Values represent the means of three independent experiments (each done in duplicate) ± standard deviation. Asterisks on the bars indicate that mean values were statistically different from the control, while asterisks on the dashes indicate differences among the same treatment at different time points (**** *p* < 0.0001).

Kefir products are considered predominantly lactic fermentations by lactic acid bacteria and yeast [40,41]. The changes of LAB and yeasts were estimated at two time points (24 and 48 h) (Figure 5).

The LAB reached the stationary phase after 24 h, since at 48 h similar result was achieved in terms of count of colony-forming unit (CFU), but the population was significantly different with respect to control and, as such, continues to be an energy source for yeasts. On the other hand, the yeast counts gradually increased also at 48 h of fermentation in the presence of pectins. Nevertheless, the production of vitamin C did not increase compared to the level obtained after 24 h in the kefir sample supplemented with green jujubes. This result suggested that jujube pectins ensure the survival of LAB and enhanced yeast growth, without further stimulation for these to produce vitamin C. After 24 h it would seem that bacteria and yeasts reach an equilibrium symbiosis that does not bring further changes in the content of vitamin C, in the pH values, or in the amount of product kefiran. Interestingly, we did not observe any differences either in LAB or in yeast counts among the kefir samples treated with pectins from the different ripening stages. The difference of vitamin C content in the kefir samples supplemented with pectins from all three collections is influenced by the degree of methoxylation which, if elevated, inhibits the hydrolytic action of the enzymes on the skeleton of polygalacturonic acid, such as galactose source for yeasts. These results support our hypothesis regarding the crucial role of pectins in the increased production of vitamin C. In particular, we can rationalize that all pectins stimulate the growth of yeasts during the first 24 h, but the pectins from jujubes at the first stage of ripening (PJ1), characterized by a lower methoxylation degree, act as the best substrates for yeasts, thus yielding to the highest amount of vitamin C.

## 3. Materials and Methods

### 3.1. Standards, Solvents, and Reagents

All chemicals and reagents, including a 99.7% purity ascorbic acid standard, were purchased from Sigma-Aldrich S.p.a. (Milan, Italy). Analytical grade solvents were bought from VWR International S.r.l. (Milan, Italy).

### 3.2. Milk Samples

Different commercial cow milk samples (Granarolo, Bologna, Italy) were purchased from a local market including pasteurized whole, partially skimmed, and skimmed. The milk samples were analyzed to establish the one with less or no vitamin C content to be used in the heterolactic fermentation. Screening of commercial milks for the determination of the vitamin C content was carried out by HPLC analyses.

### 3.3. Kefir Grains

Kefir grains were obtained from Kefiralia (Arrasate, Gipuzkoa, Spain) and were composed of 10^9^ CFU/g of LAB (*Lactococcus lactis* subsp. *lactis*, *Lactococcus lactis* subsp. *lactis biovar diacetylactis*, *Lactococcus lactis* subsp. *cremoris*, *Leuconostoc mesenteroides* subsp. *cremoris*, *Lactobacillus kefyr*), *Candida kefyr,* and *Saccharomyces unisporus* subsp., as declared by the producer. Microorganisms exist in a matrix composed of polysaccharide, referred to as kefiran [42,43]. The grains were washed three times with deionized water and then were placed in a nonhermetically sealed glass container and were grown at room temperature in 1 L of milk, without stirring. The medium was changed daily. Kefir grains to be used were separated from kefir-fermented milk by filtration with a plastic sieve and washed with cold running water.

### 3.4. Plant Materials

**Figure 6:**
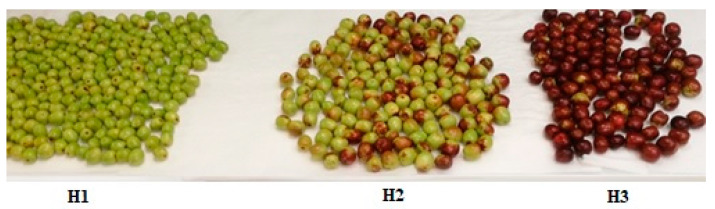
Division of harvesting stages (H1 to H3) according to appearance of jujube fruit. Harvesting stages of jujube fruits were characterized by different colors and sizes: H1, green stages; H2, yellow and green stage with mahogany-colored spots; H3, all-red ripening stage.

The jujube fruits were harvested in the locality of Rombiolo (latitude: 38°35′34”08 N, longitude: 16°0′9”00 E, Calabria, Southern Italy), in September–October 2017. The drupes were picked up from the same area three times, at a distance of 15 days from each other. The fruits, which have a round shape, changed dimensions and colors during this harvest period (Figure 6). The thin, edible skin was green in color at the first harvesting (H1), when the fruits are immature, but as ripening continued, the skin color progressed from a yellow-green stage with mahogany-colored spots (second harvesting, H2) to half red-half creamy. The fruits collected at the third harvesting (H3, almost completely red) had a crisp texture. This aspect indicated that pectin-depolymerizing enzymes, such as polygalacturonases, had not yet acted on the cell walls of the fruit, causing its softening. Fruit size was calculated by averaging the measurements of 20 randomly picked fruits. In H1 fruit length and diameter were 19 ± 1 and 4.7 ± 0.5 mm, respectively; in H2, 19.7 ± 0.4 mm of length and 6.5 ± 0.2 mm of diameter; and in H3, 21.2 ± 0.7 mm of length and 7.8 ± 0.2 mm of diameter. The fruits at all harvesting periods were freeze-dried (Telstar freeze-dryer, model Cryodos, Terrassa, Spain), followed by removing of the seeds. Dried jujubes were ground to a fine powder using a 60-mesh screen (particle size equal to 250 µm) and stored at −20 °C, before the extraction. All extractions were performed in triplicate using three samples selected for each harvesting period. The results are expressed on a dry weight (DW) basis.

### 3.5. Extraction of Pectins

Pectins from jujubes were extracted following a previously reported method [44]. Jujube flour (2 g) was suspended in 10% (*w/v*) citric acid solution (sample-to-solution ratio 1:20 *w/v*) adjusting the pH to 2. Afterwards, the mixture was kept under reflux at 90 °C with magnetic stirring for 1 h. After cooling, the mixture was centrifuged at 5000× *g* for 30 min (Universal 320, Hettich Zentrifugen, Merck, Italy). Then, absolute ethanol (1:1 *v/v*) was added to the supernatant and kept at 4 °C for 16 h in order to precipitate the pectins. The precipitate was recovered after centrifugation, washed with absolute ethanol, solubilized in distilled water, treated with acetone in the ratio of 1:1, and kept at 4 °C for 12 h to remove undesired pectin color [26]. The precipitated pectins were isolated by centrifugation at 5000× *g* for 15 min and were dried under vacuum. Extractions were performed in triplicate and data of pectin content recovered are expressed as means ± standard deviation. The pectins from jujubes of the first, second, and third harvestings were designated as PJ1, PJ2, and PJ3, respectively. All samples were characterized by FTIR and SEM.

### 3.6. FTIR Spectroscopic Analysis

FTIR spectroscopy of isolated PJ samples was carried out using a Bruker ALPHA FTIR spectrometer (Billerica, MA, USA) with KBr method in the wavenumber range of 4000–400 cm^−1^ [44]. Dried samples were ground in an agate mortar with KBr in 1:40 ratio and pressed into pellets. Twelve scans were performed with a resolution of 4 cm^−1^. All measurements were acquired in triplicate.

### 3.7. Scanning Electron Microscopy

The morphological analysis of the samples was acquired by a scanning electron microscope (SEM) (Field Emission SEM FEI Quanta 200, Thermo Fisher Scientific, Hillsboro, OR, USA) and Electron Probe Micro Analyzer (EPMA)-JEOL-JXA 8230t (Kyoto, Japan).

Before analysis, the surface of the samples was coated by a 5-nm-thick layer of carbon, using a Carbon Coater QUORUM Q150T-ES. The SEM analysis conditions were the following: high voltage (HV): 15 kV; probe current: 10–20 nanoAmpere (nA); working distance: 11 mm; image: back-scattered electrons’ technique (BSE) signal; detector image: Solid state detector (SSD), Everhart–Thornley detector (Scattered electron, SE); image size: 2560 × 1920 pixel.

SE signal was used to acquire morphological pictures while BSE signal was used for crystal observation.

The WDS analysis conditions were the following (Spectrometers WDS XCE type and X type): HV 15 kV, probe current 10 nA, and working distance of 11 mm. For WDS analysis, all experiments were carried out at room temperature (22 ± 1 °C) and, for each sample, three points were analyzed to determinate the average value for N, C, and O content.

### 3.8. Determination of Esterification Degree

The esterification degree of pectins is one of the most important properties of pectins, determined by the potentiometric titration method (DE) and confirmed by the instrumental FTIR method (DM).

#### 3.8.1. Titration Method

The esterification degree of pectins was determined by titrimetric method, as previously described [45]. Dried pectin (20 mg) sample was weighed in a bottle for titration, wetted with ethanol, dissolved in 5 mL of distilled water, and heated at 45 °C, under magnetic stirring, until the pectin dissolved completely. The resulting solution was added of 3 drops of phenolphthalein and titrated with 0.01 N NaOH till the solution color changed to pale pink (V1). This initial titration volume indicated the number of free carboxy groups.

Then, polygalacturonic acid was neutralized by adding 3 mL of 0.01 N NaOH followed by stirring at room temperature for 2 h in order to saponify the esterified carboxy groups of the polymer. NaOH was neutralized by adding 3 mL of 0.01 N HCl to the solution and then the excess HCl was titrated with 0.01 N NaOH after the addition of three drops of phenolphthalein. The sample was shaken until pale-pink color appeared, and the titration volume was recorded (V2, indicating the number of esterified carboxy groups). The DE was calculated from the following formula:

% DE = [V2 (mL)/V1 (mL) + V2 (mL)] × 100.(1)

#### 3.8.2. Instrumental FTIR Method

In this methodology, the degree of methoxylation (DM) was determined using the FTIR spectra recorded for the characterization of pectins [45]. In particular, the bands at 1748 cm^−^^1^ (arising from the ν C=O from ester group) and at 1647 cm^−^^1^ (due to the ν COO^−^ from the carboxylate group) were used to determine the % DM, according to the following equation:

%DM = [A1748 cm^−1^/(A1647 cm^−1^ + A1748 cm^−1^)] × 100. (2)

### 3.9. Heterolactic Fermentation Procedure

Kefir samples were prepared at two different fermentation times (24 and 48 h) by inoculating whole cow milk with kefir grains. Kefir grains (1 g) were placed in a glass container, in which 10 mL of cow milk was dispensed. The glass container was not hermetically closed by para film, on which holes were made by micro-needle device (fermentation produces CO_2_). Fermentation was carried out at room temperature (approximately, 20–25 °C), in aerobic conditions, and without shaking. Fermented milk with kefir grains (named as 0 h sample) was used as a control. The same protocol was applied to the milk samples containing kefir grains supplemented with pectins from jujubes of all harvesting (0.25 mg·mL^−1^): PJ1K (kefir supplemented with pectin from jujube of first harvesting), PJ2K (kefir supplemented with pectin from jujube of second harvesting), and PJ3K (kefir supplemented with pectin from jujube of third harvesting). Three independent, biological replicates were carried out for each sample. The fermentation product (i.e., kefir) was separated from the kefir grains by filtration with a plastic sieve. The pH was measured in all supernatants with an electrode pH-meter (Hanna Instruments) during fermentation (0, 24, and 48 h). Kefir grains in all fermented samples were weighed at the end of the experiment, after centrifugation at 3000 rpm for 10 min at 4 °C.

### 3.10. Vitamin C Extraction and HPLC Conditions

Vitamin C contents of whole milk, kefir, and pectin-supplemented kefirs were determined by HPLC analyses, as described previously [45]. Milk and kefir supernatants (2 mL) were mixed with 1.25 mL of acetic acid, diluted with distilled water (10 mL), vortexed for 10 s, and centrifuged at 5000× *g* for 10 min to separate coagulated caseins. The supernatants were filtered through sterile 0.45-μm pore-size filters (Millipore, Millex-GV, Darmstadt, Germany), to remove cells released from the kefir grains, into amber vials and injected directly into the HPLC system.

HPLC analyses were carried out using a high performance liquid chromatography-diode array detector (HPLC-DAD) system (Shimadzu, Kyoto, Japan), equipped with two LC-20A pumps and an SPD-M20A DAD detector; the column used was a mediterranea SEA 18 (Teknokroma, Barcelona, Spain) (250 mm × 0.46 mm_id_, 5-µm particle size). The analyses were carried out at a constant flow rate of 0.7 mL·min^−1^ using a mobile phase of H_2_SO_4_ in deionized water at pH of 2.5. The injection volume was 20 µL, the detection wavelength 254 nm, and the run time was 40 min. Data were integrated and analyzed using the Shimadzu Class-VP Chromatography Laboratory Automated Software system. Quantitative analysis was carried out by the external standard method using standard solutions at a concentration range of 10–500 µg·mL^−1^. Determination of coefficients obtained from these calibration curves were linear over the range studied (R^2^ = 1).

### 3.11. Bacterial and Yeast Counts

Lactic acid bacteria and yeast counts were carried out in duplicate on kefir samples added (or not, control) with pectins from jujubes at different ripening stages, at two time points (24 and 48 h), according to a known protocol [11]. Samples were diluted with sterile saline solution (1:10,000) and plated on sterile bromocresol purple (BCP) agar (Condalab, Madrid, Spain) at 37 °C for 3 days to determine LAB, or on sterile potato dextrose agar (Condalab, Madrid, Spain) at 24 °C for 5 days to determine yeast counts.

### 3.12. Statistical Analysis

The experimental data of methoxylation degree (DM and DE), pH, vitamin C content, and LAB and yeast counts of kefir samples during fermentation are presented as mean values ± standard deviations. Each experiment was replicated three times. Statistical differences among all prepared kefirs (control and PJ1K, PJ2K, PJ3K) were evaluated by two-way ANOVA followed by Holm–Sidak’s multiple comparisons’ post hoc test. The significance was established at *p* values < 0.05 (*), *p* < 0.01 (**), and *p* < 0.0001 (****).

## 4. Conclusions

In the present work, we characterized the pectins from jujubes at three different degrees of ripeness (PJ1, PJ2, and PJ3) by means of FTIR and SEM. Pectins from jujubes at the first stage of ripening (PJ1) were characterized by the lowest methoxylation degree, as it was confirmed by instrumental as well as titrimetric methods. The addition of all pectin samples to milk containing kefir grains (the control) influenced the acidity and vitamin C amount in kefir beverages, prepared by inoculating whole cow milk, not containing vitamin C, with kefir grains. Moreover, the amount of vitamin C content in kefir was found to depend on the ripening stage of the fruits as kefir obtained by the addition of PJ1 (PJ1K) resulted in a quantitatively higher vitamin C level compared to all the other samples (PJ2K and PJ3K), after 24 and 48 h fermentation. In particular, the amount of vitamin C in PJ1K was 2.3 times higher than that recorded in the control. This result fit well with the pH value observed, which was the lowest value among all kefir samples (3.73 ± 0.02). To clarify the role of microorganisms on the production of vitamin C and pH values of the samples during fermentation, an estimation of LAB and the yeast population at 24 and 48 h was carried out. The yeast counts gradually increased also at 48 h of fermentation in the presence of all pectins. The results supported the hypothesis that all pectins stimulate the growth of yeasts during the first 24 h, possibly supplying them galactose as a substrate. No differences were observed either in LAB or in yeast counts among the kefir samples treated with pectins from the different ripening stages. The difference of vitamin C content in the kefir samples can be influenced by the degree of methoxylation which, if elevated, inhibits the hydrolytic action of the enzymes on the skeleton of polygalacturonic acid. Then, pectins from jujubes at the first stage of ripening (PJ1), characterized by a lower methoxylation degree, act as the best substrates for yeasts, thus yielding the highest amount of vitamin C. Hence, this study showed that pectins from jujubes could be used to increase the amount of vitamin C in kefir, although the final amount is far from being close to the recommended dietary intake. Further research is needed to possibly overcome this limitation.

## Figures and Tables

**Table 1:** Wavelength Dispersive X-ray Spectroscopy (WDS) measurements of the compositions in wt.% of PJ1, PJ2, and PJ3.

Samples	N	C	O
PJ1	1.24 ± 0.02	46.17 ± 0.09	7.98 ± 0.88
PJ2	1.65 ± 0.18	63.61 ±.0.94	12.93 ± 0.97
PJ3	1.92 ± 0.14	69.87 ± 0.84	12.76 ± 0.76

**Table 2:** Kefir weight in all fermented samples, expressed in g.

Samples	0 h	24 h	48 h
Control	1.01 ± 0.01	1.65 ± 0.01	1.95 ± 0.01
PJ1	1.01 ± 0.01	2.60 ± 0.03	2.10 ± 0.04
PJ2	1.04 ± 0.06	1.60 ± 0.02	1.46 ± 0.05
PJ3	1.01 ± 0.01	1.40 ± 0.01	1.51 ± 0.06

Values represent the mean of three independent measurements (each done in triplicate) ± standard deviation.

## Abbreviations

H1	jujube at the first harvesting
H2	jujube at second harvesting
H3	jujube at the third harvesting
PJ	pectins from jujubes
PJ1	pectins from jujubes of first harvesting
PJ2	pectins from jujubes of second harvesting
PJ3	pectins from jujubes of third harvesting
PJ1K	kefir supplemented with pectin from jujube of first harvesting
PJ2K	kefir supplemented with pectin from jujube of second harvesting
PJ3K	kefir supplemented with pectin from jujube of third harvesting
FTIR	Fourier Transform Infrared Spectroscopy
DE	esterification degree determined by titration method
DM	esterification degree determined by instrumental FTIR method
SEM	Scanning Electron Microscopy
WDS	Wavelength Dispersive X-Ray Spectroscopy
HM	high-methoxyl
LM	low-methoxyl
